# Enabling Remote Elderly Care: Design and Implementation of a Smart Energy Data System with Activity Recognition

**DOI:** 10.3390/s23187936

**Published:** 2023-09-16

**Authors:** Patricia Franco, Felipe Condon, José M. Martínez, Mohamed A. Ahmed

**Affiliations:** Department of Electronic Engineering, Universidad Técnica Federico Santa María, Valparaíso 2390123, Chile; patricia.franco@usm.cl (P.F.); felipe.condon.13@sansano.cl (F.C.); jose.martinez@usm.cl (J.M.M.)

**Keywords:** activity detection, appliance recognition, load monitoring, machine learning, non-obtrusiveness, remote healthcare, simultaneous detection

## Abstract

Seniors face many challenges as they age, such as dementia, cognitive and memory disorders, vision and hearing impairment, among others. Although most of them would like to stay in their own homes, as they feel comfortable and safe, in some cases, older people are taken to special institutions, such as nursing homes. In order to provide serious and quality care to elderly people at home, continuous remote monitoring is perceived as a solution to keep them connected to healthcare service providers. The new trend in medical health services, in general, is to move from ’hospital-centric’ services to ’home-centric’ services with the aim of reducing the costs of medical treatments and improving the recovery experience of patients, among other benefits for both patients and medical centers. Smart energy data captured from electrical home appliance sensors open a new opportunity for remote healthcare monitoring, linking the patient’s health-state/health-condition with routine behaviors and activities over time. It is known that deviation from the normal routine can indicate abnormal conditions such as sleep disturbance, confusion, or memory problems. This work proposes the development and deployment of a smart energy data with activity recognition (SEDAR) system that uses machine learning (ML) techniques to identify appliance usage and behavior patterns oriented to older people living alone. The proposed system opens the door to a range of applications that go beyond healthcare, such as energy management strategies, load balancing techniques, and appliance-specific optimizations. This solution impacts on the massive adoption of telehealth in third-world economies where access to smart meters is still limited.

## 1. Introduction

Current research in ambient assisted living (AAL) has been oriented to assess the feasibility of using technology in healthcare practices. AAL utilizes available technology across different domains such as computer science, engineering, medicine, and social sciences to identify human activities and provide medical insights, commonly referred to as telehealth. Additionally, AAL technologies provide two main types of monitoring: preventive, which forewarns patient risks by analyzing activities of daily living (ADLs), and responsive, which reacts to events such as falls, alarms, and patients leaving their home [1]. This can be achieved through the analysis of the data provided by home devices using smart appliances, wireless networks, software applications, smart meters, and medical sensors [2]. In other words, smart energy data can be used to provide unobtrusive health monitoring [3].

The report of the Smart Future of Healthcare Workshop in February 2020 (see https://2020health.org/publication/smart-future-of-healthcare/ (accessed on 20 May 2022)) examined different ways in which smart energy data can be used in remote healthcare. According to the authors, applications are collected in three broad domains: ambient assisted living support, population-level screening and support, and self-monitoring. The first clinical trial using smart energy data for telehealth was conducted in 2016 [4]. Since then, the potential benefits of smart energy data in supporting health and care systems are of increasing interest due to the massive adoption of smart meters and other smart grid technologies in a growing number of countries worldwide.

A potential source of smart energy data comes from the residential sector [5]. In smart homes, home energy management systems (HEMSs) are expected to enable efficient service management, provide customers with comprehensive internal information exchange functions, and help family members maintain continuous contact with the outside world [6]. The application of the smart home concept and HEMSs aims to facilitate the user’s operation and management of household appliances to achieve automation and optimized operation [7]. To establish such a management system, the first step is to identify and monitor the energy consumption of the main electrical appliances in the home. This is known as load monitoring [8]. Common loads of interest are washing machines, dishwashers, vacuum cleaners, electric vehicles (EVs), and heating, ventilation, and air conditioning (HVAC) [2,9]. Using data collected from electricity readings, technology can accurately identify the use of individual electrical devices in the home and routine behaviors of people to detect when anomalies occur [10,11].

**Figure 1:**
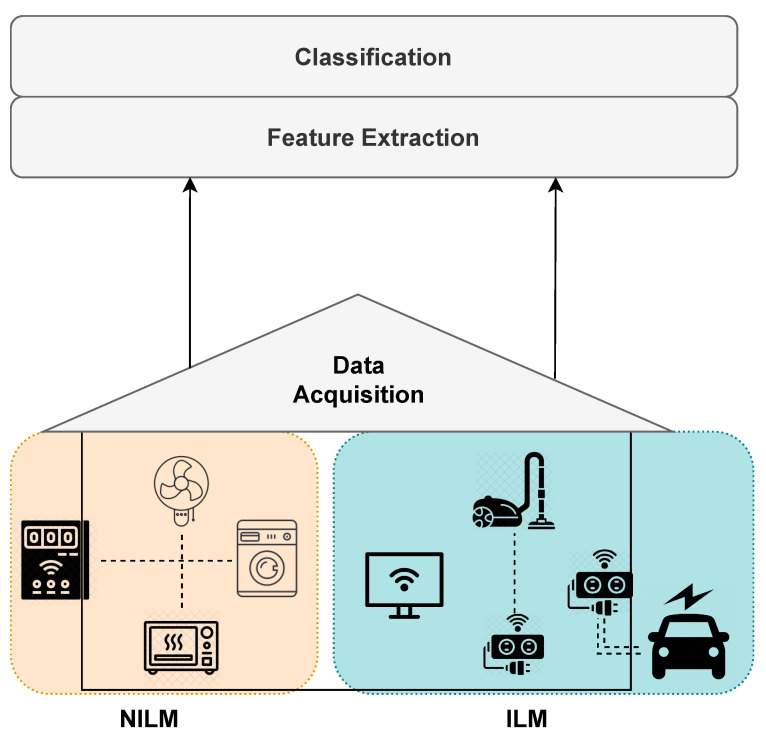
Schematic diagram of load monitoring methods. On the left, software-based (NILM), and on the right, hardware-based (ILM).

Load monitoring techniques are classified into two main groups, as shown in Figure 1: software-based methods (non-intrusive load monitoring (NILM)) and hardware-based methods (intrusive load monitoring (ILM)). Both categories consist of a data acquisition stage, involving appliances and metering devices, and an analytics part, commonly consisting of two stages: feature extraction and classification. The last two processes are usually carried out in a middleware technology, being a local/remote server. Machine learning (ML) techniques are employed in both monitoring methods, especially for classification. The main difference between the two methods lies in the acquisition stage, since NILM uses smart meters as a single point of sensing, while ILM implies multiple sensors. Furthermore, ILM has two subcategories: one is based on metering devices (e.g., smart plugs attached to home appliances) known as distributed sensing, and the other is based on smart appliances. Smart appliances are devices with built-in capabilities to monitor and report their consumption [2,12]. Both NILM and ILM need a communication network to allow data exchange between local devices (appliances, meters, and home gateway) and the middleware where the analytics stage takes place. Therefore, load monitoring systems can be represented as an internet-of-things (IoT) platform able to support activity recognition and a wide variety of services.

According to [1], research activities in the medical field are interested in ILM and NILM approaches and their applications in delivering home healthcare services. Compared to hardware-based methods, NILM offers easier installation, since it only needs one point of sensing, but achieves less accurate results and adds more difficulty to implementation in practical scenarios. The aggregated signal can be very noisy and few appliances can be detected depending on the sampling frequency [8]. Even with advanced artificial intelligence (AI) algorithms, only a few major appliances can be monitored (e.g., ovens, washing machines, HVACs, EVs). Furthermore, the performance of NILM on different datasets remains inconclusive in terms of device types and metrics used [13]. On the other hand, ILM methods offer greater reliability than NILM but require the installation of more equipment, which results in an increase in the costs [14,15]. However, with the growing popularity of smart plugs in the market, this situation has changed. These metering devices are small, compact, compatible with traditional plug-in sockets, and accessible in most countries. They combine various technologies and address all aspects for effective load monitoring. In contrast, current smart meter technology has problems with changes in energy consumption, privacy and security of the metering data, and the management of collected data. Therefore, distributed sensing using smart plug devices becomes a potential solution that can unobtrusively improve the process of efficient low-cost load monitoring, bringing the possibility of converting traditional devices to be smart [16]. This allows ADLs and routine behavior patterns of householders to be identified, obtaining valuable information not only for health but also for energy efficiency, user satisfaction, and sustainability of homes. However, the implementation capabilities of these systems in practice are quite limited, since the absence of reliable evidence prevents any definitive guide or recommendation for their operation in a real environment. The main challenge is to develop a platform able to work in real time identifying simultaneous ADLs (i.e., multiple activities at the same time).

Specifically in Chile, regulations exist for the use of smart meter technology but access to smart appliances is still limited due to economic reasons. On the other hand, according to the results of the 2017 census (see https://www.ine.gob.cl/estadisticas/sociales/censos-de-poblacion-y-vivienda (accessed on 10 June 2023)), around 2.4 million people are living alone. Telemedicine in Chile is mainly oriented to teleconsultations in ambulatory, hospitalization, and urgency care, telereports, and explicit health guarantee networks (GES), according to the national telehealth program [17]. Therefore, the introduction of smart energy data, ambient assisted living technologies, load monitoring, and the classification of activities of daily living will bring significant opportunities to improve the quality of life and remote healthcare services of Chilean society.

In this work, we propose the design and implementation of a smart energy data with activity recognition (SEDAR) system for monitoring the main loads inside a household and extracting routine behavior patterns of consumers, which can significantly impact independent living and social care. We develop a platform that benefits from edge technologies to provide relevant behavioral information of users living independently. We consider smart energy data generated through the interaction with existing household appliances as the only source of information. This allows the identification of low- and high-power devices, such as the television set (TV) and the heater, without interfering with the user’s routine. Therefore, privacy and acceptability concerns are mitigated through the use of smart plugs installed across the house. Every appliance can be connected to any plug, thus, giving the system flexibility and making the house smart. Using ML and statistical analysis techniques, the system allows appliance usage frequency, activity distribution, and average inactivity periods to be collected to build a user profile.

We deployed the real system in a laboratory environment at Universidad Técnica Federiso Santa María (UTFSM), Valparaiso, Chile, overcoming the implementation issues of previous approaches. The proposed platform can be used for different purposes, either as a family-oriented tool for remotely monitoring the elders living alone, without intervention in their daily routines, or as a comprehensive solution for monitoring the energy consumption of a household, coupling with an HEMS. In Chile’s context, the proposed system can help to overcome the emerging challenges in telehealth programs targeting a massive deployment of remote healthcare systems for elderly care.

To the best of our knowledge, no prior research has tackled the need for reliability in the data source by presenting a solution utilizing smart plugs, and no similar solution currently exists in Chile. Furthermore, no previous studies have addressed the uncertainty associated with evaluation metrics, such as the accuracy, by proposing various preprocessing techniques and more complex ML models. In addition, there has been a lack of practical implementation of load monitoring and remote healthcare systems, particularly in edge-based settings. The contributions of this work are summarized as follows:

- We designed and validated an IoT platform in a real scenario to unobtrusively perform load monitoring and activity recognition (ADLs), aiming to enable remote elderly care in Chile.
- The proposed system achieves near real-time operation by accurately identifying both low- and high-power-consumption devices, overcoming the limitations of NILM solutions in this regard.
- The proposed system is capable of recognizing activities being simultaneously performed, and showing the information to users in a friendly manner through a dashboard interface.
- The proposed system is flexible, allowing the connection of any appliance independently of the plug, making it adaptable to different devices.

The rest of this paper is organized as follows: in Section 2, we present a comprehensive study of state of the art for IoT and ML solutions regarding in-home monitoring and elderly care. In Section 3, we discuss previously used techniques, their limitations, and the novelty of the proposed strategy. In Section 4, we give detailed information about the proposed system. Next, in Section 5, we describe the processing applied to the collected data. In addition, in Section 6, we explain the experiments performed and the results obtained. Furthermore, in Section 7, we analyze the advantages and disadvantages of the system. Finally, in Section 8, we arrive at conclusions and present the guidelines for future work.

## 2. Related Work

  In recent years, a significant part of the research community has been interested in the advances in the IoT, sensing, and communication technologies for telehealth. Two main views can be distinguished when analyzing related work in this regard: applications for hospital/medical services (medical oriented) and applications for family members (family oriented). Both approaches aim to monitor people remotely by identifying routine behavior patterns and if and when anomalies occur. The main difference between medical- and family-oriented applications lies in the type of anomalies or information given by the proposed application. Although medical services show a more detailed report that can help treat a specific disease, family-oriented applications show an overall context of patient/person behavior.

### 2.1. Medical-Oriented Applications in Research

To fulfill medical applications, wearable sensors have been preferred due to their capabilities in diagnostic and monitoring applications. These devices are capable of gathering physiological and movement data of patients, providing status monitoring [18]. The authors of [19] reviewed low-cost and accessible IoT technologies available for biomedical engineers, presenting a comprehensive insight into the technical specifications of sensing devices, including wearable sensors. A different solution is presented in [20], where the authors designed a distributed platform to monitor the patient’s movements and status during rehabilitation exercises. However, since these approaches required the installation of devices near the patients, in some cases attached to their clothes, inconveniences to everyday life can be introduced [3]. Similar problems occurred in [21], in which the authors used a camera to record and employed computer vision techniques to perform the recognition. This raised privacy concerns, and hence, a low opportunity for a massive adoption of the system. To overcome such privacy issues and avoid disrupting the normal routine of people, in [1], the authors presented an NILM-based system capable of recognizing anomalous behavior in dementia patients. Machine learning algorithms were trained with data collected during a real-case scenario campaign. This approach facilitated the detection of specific ADLs in an unprecedented manner without incurring undue burden on the cognitive demands of patients or cost. However, since NILM relies solely on smart meter data, only major appliances were detected, including the kettle, microwave, toaster, electric oven, and washing machine. On the other hand, the authors of [22] carried out a pilot study for in-home monitoring of patients with Parkinson’s disease (PD), focusing on house activity and time in bed. A device was installed in the bedroom of seven ambulatory individuals. The device, called Emerald, was able to passively detect routine activity of patients using radio waves, however, only certain points of interest could be monitored. The authors of [23] proposed a platform which gave promising results, especially in fall detection, but it was based on flexible non-contact triboelectric sensors (NCTSs). A similar cloud-based platform for providing support to the healthcare medium through load-monitoring-based solutions was discussed in [24]. Apart from being based on NILM techniques, no practical implementation was provided.

Although previous research has successfully achieved human activity recognition, allowing the monitoring of patients with several illnesses, the results are still inconclusive in terms of non-activity detection. This means, how to differentiate whether if the patients are sleeping, not at home, or if some other alarming situation occurs. In addition, various authors have highlighted the lack of progress and reliability as the main difficulty in the development of remote healthcare applications. This is mainly due to the fact that government and institutional funding opportunities have not recognized the specific multi-disciplinary requirements for rigorous clinical research involving smart energy data and ML.

### 2.2. Family-Oriented Applications in Research

Family-oriented applications have mainly targeted elderly people living alone in such a manner that family members can remotely monitor their beloved ones (in-home monitoring) by analyzing their power consumption. Recent innovative solutions in remote healthcare have reaffirmed this idea. In [8,25], the authors used NILM techniques to recognize ADLs. Specifically, in [25], the authors compared results in houses with single occupants and multiple members. Their model succeeded in carrying out rough monitoring of most elderly people on a large scale, however, some adjustments had to be made in order to improve the accuracy. In the case of [8], the system struggled with simultaneous detection, being only capable of recognizing one activity at a time. Another example is [26], in which the authors presented Smart Home Control, an intelligent platform to offer fully customized automatic control schemes and perform an analysis of historical records and detect residents’ behavior patterns through IoT and ML. Nevertheless, this system conveyed challenging problems related to the difficulty in interpreting the results obtained by decision trees and random forest algorithms, leading to a lack of explainability in the generated models. On the other hand, the authors of [27] focused on detecting anomalies following an intrusive approach. They also used ML techniques, specifically probabilistic networks and H2O autoencoder, for identifying both activities and abnormal behavior. This system relied on a set of pre-segmented activities rather than appliance usage. The validation of such a system in a practical scenario is also missing. A similar problem arises in [28], in which the authors proposed a human activity recognition (HAR) model using a semi-supervised transfer learning algorithm, but experiments were run using a public radar-based HAR dataset. In [29], the authors presented ApplianceNet, a smart-plug-based mechanism to recognize appliances being used and residential patterns. The work was oriented to in-home monitoring, identifying six different activities of consumers. However, the work is only simulation-based, using data from five houses of the REFIT dataset, thus, a practical validation of such a system is required.

Regarding home care for elderly people, further research is required to conduct a comprehensive analysis that extends beyond the comparison of different ML models and techniques. As mentioned by the authors of [30], the implementation of such systems in real-time scenarios is still limited. Similar to medical-oriented applications, the results remain inconclusive when it comes to identifying non-activity. In the same way, the accuracy of the models varies on different datasets, leading to uncertain results. Most authors exploit the benefits of cloud-based systems, but having a centralized management may decrease efficiency in large-scale implementation scenarios. Exploring edge solutions can help to overcome this issue by having local management and remote storage [6]. However, various alternatives exist on where to put the intelligence, and the comparison of such systems with a so-called "blind" platform (i.e., without AI or intelligence) is still missing.

### 2.3. Summary

As a summary, Table 1 highlights the main aspects identified in the literature, comparing the methodologies followed by previous authors and the one proposed in this work. The first two columns contain the references and the type of paper, i.e., technical or survey. In essence, there are three different data sources as the main providers for AAL and telehealth applications, wearable sensors, cameras or visual information, and dense sensing devices (such as smart plugs, smart meters, and others), which are used in the data acquisition stage of both ILM and NILM solutions. These characteristics are shown in the next four columns of Table 1. Also, we summarize the target domain which the given study can help to mitigate or monitor according to the researchers. These are directly related to medical- and family-oriented applications. As can be seen, this work proposes ILM techniques for recognizing common ADLs, which provide the system with higher reliability compared to those based on NILM, and with less obtrusiveness than those based on wearable sensors or cameras. Additionally, the system offers advantages as both an in-home monitoring system for tracking total and individual appliance power consumption, and as a remote elderly care solution capable of generating a user profile based on behavior patterns such as appliance usage frequency, activity distribution, and average periods of inactivity.

## 3. Methodology

  Useful data for in-home monitoring can be provided by three main sources related to the physical devices used to collect data: wearable devices, cameras, and other sensing devices. These sources have been commonly classified as wearable sensors and non-wearable sensors [32]. Wearable sources includes those devices which need to be carried by the user in order to sample vital information. Devices such as gyroscopes, accelerometers, and radio-frequency identification (RFID) tags are part of this category. As a special type of wearable device, smart phone-based applications are also considered since the user must carry the smart phone to collect the data. Wearable devices may bring discomfort and privacy issues which is not convenient in many cases. On the other hand, non-wearable sources include two subcategories, vision-based approaches and dense sensing, which do not require any user involvement. Vision-based solutions consist of infrared (IR), depth, or common cameras installed in the vicinity of the household to monitor the user’s activities. Although this technique gives detailed information, it has significant constraints regarding privacy of occupants and the complexity of the analytics algorithms. In addition, it is required that users or occupants be placed in the line of sight of the camera, which in many occasions is difficult to guarantee. Conversely, dense sensing involves any other sensors (RFID, motion, temperature, smart plugs, smart meter) which can be deployed in the household, and they can provide useful data to monitor user activity. A primary advantage of this method hinges on it not requiring any extra user intervention or physical contact other than regular activities [6,32].

Dense-sensing-based human activity recognition techniques have been categorized into three main groups: action-based, interaction-based, and motion-based [43]. Depending on the type of sensor deployed to collect data, the appropriate category will be selected. For example, interaction-based solutions consider human–object interactions while motion-based sensors include movement tracking and motion sensors. In particular, action-based approaches have proven to be a reliable option in healthcare applications since they involve ADLs and AAL. These solutions benefit from smart energy data to monitor household occupants without requiring the installation of special equipment. Both NILM and ILM methods have been widely used in this regard, offering state-of-the-art results in experimental scenarios [1,22]. However, non-intrusive methodologies face several limitations regarding standardization, detection of non-activity, and widespread adoption, owing to the reliance on smart meters [8,10].

Particularly in Chile, the slower integration of smart meter technology can be attributed to several factors:

- Cost concerns: high installation costs deter utilities and consumers.
- Infrastructure challenges: upgrading existing infrastructure is a complex and expensive task.
- Lack of awareness: consumers might not fully understand the benefits of smart meters.
- Privacy and security: concerns about data privacy and security hinder adoption.
- Regulatory hurdles: complex regulatory processes delay widespread roll-out of smart meters.
- Utility resistance: utilities might resist operational changes.
- Financial constraints: economic challenges impact adoption decisions.
- Vendor availability: limited supply chain options currently exist in the country.

On the other hand, access to smart plugs in Chile aligns well with their potential for in-home monitoring and remote elderly care. Competition among vendors, utility initiatives, and established import and distribution networks further contribute to their prevalence. The ease of integration and consumer awareness of energy-saving technologies have propelled the popularity of smart plugs. This accessibility not only supports Chile’s sustainability goals but also enables a more detailed activity profile of consumers, since both high and low power consumption appliances can be monitored.

**Figure 2:**
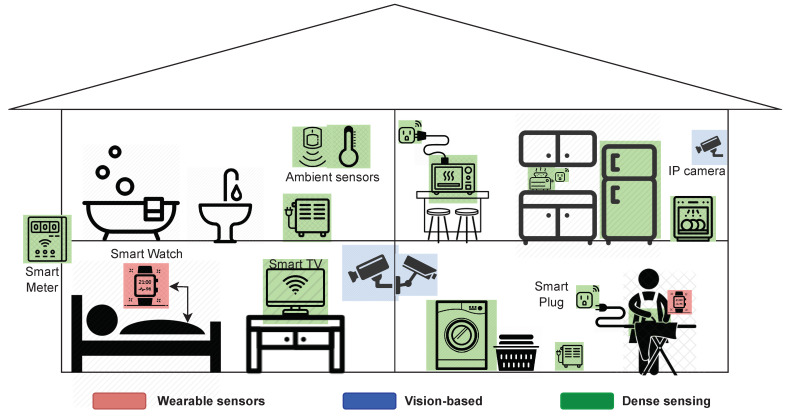
Basic schematic of enabling technologies and techniques for activity recognition and in-home monitoring.

A schematic of all of the enabling technologies for activity recognition and in-home monitoring is depicted in Figure 2. The devices involved in each category are represented in different colors. Wearable devices (smart watch) are colored red, vision-based equipment (IP camera) is colored blue, and dense sensing is colored green. Both ILM and NILM techniques are based on dense sensing for data acquisition. As ILM offers higher reliability than NILM, and access to smart plugs is increasing in Chile, we selected ILM to carry out this work. However, the total power consumption is also considered in the data analysis and can be visualized along with the individual appliance consumption breakdown.

The ILM technique is defined as a set of metering devices denoted by D attached to home appliances. Every smart plug d∈D sends univariate time-series readings $r_{d}$ at each time instant t [29]. This measurement is continuously repeated after an interval of length Δ*t*. Then, the time-series sample for a smart plug d is represented as a sequence of length N, as represented in Equation  (1):

(1) $$\begin{array}{c} R_{d}={\left(r_{d}\left(t_{1}\right),r_{d}\left(t_{2}\right),r_{d}\left(t_{3}\right);\ldots ;r_{d}\left(t_{N}\right)\right)}^{T};\forall d\in D, \end{array}$$

in which $t_{i+1}=t_{i}+\Delta$*t* and $R_{d}$ is the transpose of the *d*th smart plug sample. Each smart plug *d* is assumed to be independent, thus, it is possible to analyze a single smart plug and repeat the analysis for the rest of the plugs in D. Then, the system is simplified as in Equation (2), so that $r_{d}=r$ and sequence with length $t_{1}=t$, where t∈{1,2,3…N} [29].

(2) $$\begin{array}{c} R={\left\{r\right\}}_{t=1}^{N} \end{array}$$

Therefore, to develop such a load monitoring and activity recognition system (action-based dense sensing), in addition to appliances and metering devices, further processing is needed. The task is to first identify the appliances being used and then infer an activity according to the labels assigned [10]. Therefore, it is reasonable to think of the structure of such a system from an IoT perspective.

Usually, three- to five-layer architectures are necessary when considering appliances, metering devices, communication technologies, middleware technologies, and data visualization [6]. Four-layered architectures are commonly an extension of the three-layered architectures, since the communication network layer separates the home area network and remote communication network, which are also defined within middleware technologies in many cases. In some scenarios, a customized data visualization layer is included and oriented specifically to the target application [31]. However, having a smaller number layers can offer advantages such as simplicity, ease of implementation, and reduced overheads [48]. A critical aspect to consider is security. While some authors argue that security should be ensured at every layer [49], others propose the inclusion of an additional layer specifically dedicated to security concerns [26]. Several threads, from physical attacks to malware infection, need to be handled to provide a reliable monitoring service.

## 4. Design and Implementation of the Proposed SEDAR System

**Figure 3:**
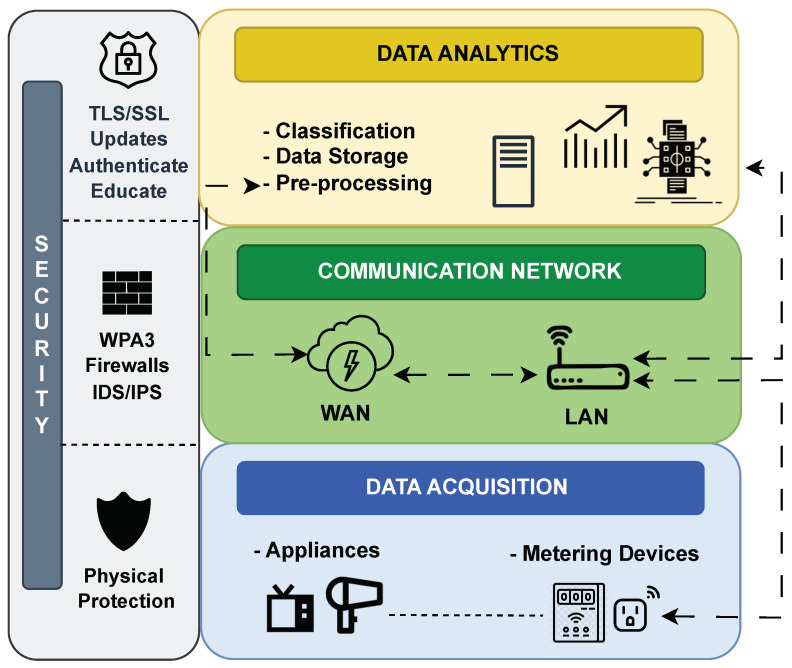
Architecture of the proposed SEDAR. Three-layer structure: data acquisition, communication network, and data analytics. LAN: local area network; WAN: wide area network.

  The proposed architecture, illustrated in Figure 3, is structured into three layers, each playing a vital role in the system’s overall functionality.

- The lower layer, called data acquisition, encompasses physical devices such as appliances and metering devices (smart plugs). At this layer, energy transactions take place.
- Moving up, the communication network layer incorporates various network technologies available in the market for local communication. It connects smart plugs with the home gateway and establishes a connection between the home gateway and middleware.
- Next, the data analytics layer gathers a range of technologies, including ML models and preprocessing algorithms for data processing, showing this information to users through a web interface. This layer serves as a mediator between physical devices and services. The integration of a diverse array of healthcare services is possible, covering in-home monitoring, user comfort, safety, and behavior analysis.

Security measures are considered at every layer, rising as a transversal layer in the proposed architecture. For the data acquisition, physical considerations are needed. If the physical security of the devices is compromised, attackers might gain direct access to sensitive information about users. For the communication network, security includes strong Wi-Fi Protected Access 3 (WPA3) for the local network and the use of firewalls and intrusion detection/prevention systems (IDS/IPS) to the wide area network (WAN) traffic for suspicious activities and potential intrusion attempts. In the case of the data analytics layer, common security measures include authentication and access controls for the middleware technology, the use of encryption transport layer security (TLS) and secure socket layer (SSL) certificates to protect messaging, regularly updating and patching software on all components to address known vulnerabilities, monitoring traffic for unusual activities, and educating users about security best practices and potential threats to prevent social engineering attacks.

It is important to clarify that in this context, the term `’users” refers specifically to the individuals who receive the processed data, rather than the occupants of the house.

**Figure 4:**
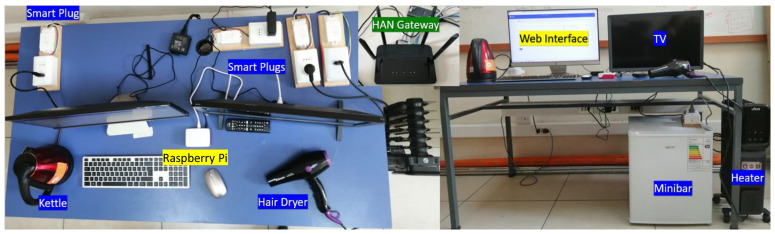
Testbed implementation in B110 Lab, Universidad Técnica Federico Santa María, Chile. Data acquisition devices highlighted in blue; home gateway highlighted in green; Raspberry Pi, and web interface highlighted in yellow. HAN: home area network.

To validate the design of the proposed architecture, a testbed was implemented in the B110 Telematics Laboratory, Universidad Técnica Federico Santa María, Valparaiso, Chile. The system’s setup is depicted in Figure 4, with labels highlighted using the corresponding layer colors assigned in Figure 3.

It is crucial to emphasize that Figure 3 encompasses all possible configurations of the system, including cloud-/edge-based setups, with or without smart meters, and the inclusion of additional services in the analytics stage. The purpose of this figure is to illustrate the functionality of each component within the layers, which remains consistent regardless of the specific configuration or setup employed. Figure 4 shows the deployed setup, representing one of the possible configurations.

### 4.1. Data Acquisition Layer

The goal in the data acquisition (DAQ) layer is to obtain load measurements at an adequate rate, aiming to identify distinctive load patterns in the following stages [50]. Therefore, in the DAQ layer, two main entities collaborate: one is household appliances and the other is metering devices. The metering devices can be installed at four different levels according to the equipment deployment granularity in the DAQ layer [6]:

- Area level: The metering devices are used to monitor household areas, measuring the consumption after the utility’s energy meter.
- Plug level: The metering devices are located next to the plugs to monitor directly appliances connected to the outlet or multi-outlet.
- Appliance level: The metering devices are embedded directly in the appliances or placed in a dedicated outlet (i.e., the outlet for a specific appliance).

**Figure 5:**
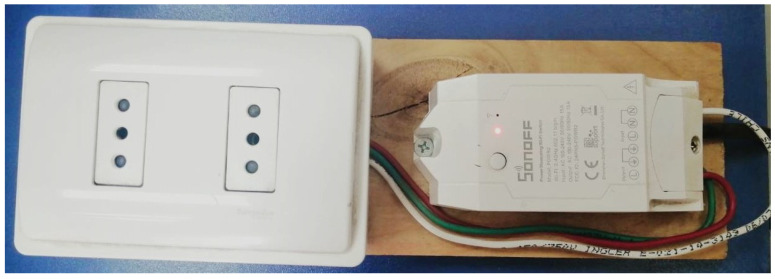
Sonoff Pow R2 integrated with conventional plug to collect data.

To develop this work, smart plug devices were installed at plug level, located next to the outlet. This means that every appliance can be connected to every plug, hence the need for labeling. Due to market availability in Chile, Sonoff Pow R2 devices were selected for use with the system. These devices are able to acquire readings from appliances, but they lack a plug. As a solution, the Sonoff Pow R2 devices were integrated into a conventional plug, as shown in Figure 5. Sonoff devices have some limitations with the proprietary firmware, not having the capability for being plug and play. To solve this issue, we installed ESPurna as firmware. This is a custom firmware for ESP8285-/ESP8266-based smart switches, lights, and sensors. It uses the Arduino core for the ESP8266 framework and a number of third party libraries. The ESPurna firmware allows control of the Sonoff devices through a web interface, called Web UI, where different parameters can be configured, such as the message queue telemetry transport (MQTT) protocol.

At this stage, a crucial parameter to consider is the sampling rate. The data sampling can be classified into two categories: high-speed sampling and low-speed sampling. Depending on the target application, the sampling rate for electricity consumption may vary. A fairly high sampling rate ranges from 1 kHz to almost 100 kHz in most cases [2,8,51]. For higher sampling rates, the identification results are more precise, typically allowing state transitions to be captured and eventually separating brands in the same category [51]. However, most commercial devices cannot achieve high-speed sampling. Furthermore, the complexity of data storage, transmission and processing for high-speed sampling is significantly increased compared to low-speed sampling [2]. In the case of this work, we set the sampling rate to 6 s, a high sampling value, used in several previous state-of-the-art studies [8,10,11] and well-known datasets [52].

Furthermore, an eGauge data logger was installed to monitor the overall power consumption of the laboratory, simulating the functionality of a smart meter within a household setting. The appliances used in this work are summarized in Table 2. According to Enel, a Chilean service provider, these are five of the most common appliances in Chile (see https://www.enel.cl/es/clientes/tarifas-y-regulacion/consumo-artefactos-electricos.html (accessed on 15 May 2023)). The columns represent the brands and models. All devices were purchased in 2022; they operate with a voltage of 220 V, and a frequency of 50 Hz according to the Chilean standard.

### 4.2. Communication Network Layer

In order to connect metering devices to an application host or service provider, a communication network must be deployed. Two types of networks need to be managed in order to implement an in-home monitoring system:

- Home area network (HAN): Inside a household, the home area network is used to provide monitoring of energy usage. This communication network carries data generated by the metering devices and home appliances to the middleware technology in which the post-processing (monitoring, control, comfort analysis, occupancy, among other applications) is performed. Examples of communication technologies include IEEE 802.3 family, power line communications (PLCs), serial communication RS-232/485, wireless networks (IEEE 802.11 family, IEEE 802.15 family, mobile field network) (GSM-based 2G, CDMA-based 3G, LTE-based 4G, NR-based 5G), and low-power networks (NarrowBand IoT, LoRa, Sigfox) [53,54].
- Wide area network (WAN): Outside the household domains, the WAN provides data exchange between smart homes and services providers, forming smart neighborhoods and cities. Furthermore, central managed solutions, such as the cloud-based load monitoring system and database servers, are accessible through this communication network.

Since this work focuses on local communication, the proposed system only considers a HAN. Wi-Fi technology was employed to enable the communication between smart plugs and the HAN gateway, as well as the edge middleware devices. To facilitate this communication, MQTT messages are transferred over the Wi-Fi network.

### 4.3. Data Analytics Layer

The data analytics layer (DAN) encompasses the middleware technologies, which can consist of a cloud-computing-based central processing mechanism and/or edge-based distributed computing intelligence. These technologies are responsible for executing and optimizing data processing strategies within the system. In these processes, AI and ML models can be deployed, enabling the system to understand the routine and life habits of multiple householders. In this way, the data can be reused, accumulated, and visualized at any time [55]. Therefore, two main tasks need to be accomplished at this stage [56]:

1. Collect data from different metering devices at the plug level through the HAN.
2. Provide monitoring and analysis of the main loads inside a household.

Feature extraction and classification techniques as part of load monitoring are crucial for the initial identification of major appliances that contribute to higher electrical consumption, and for the further development of the consumer profile, which provides useful information such as behavior patterns and other activities (ADLs) [10,11]. Major appliances are mostly used by consumers for routine housekeeping tasks such as cooking, doing laundry, or food preservation.

**Figure 6:**
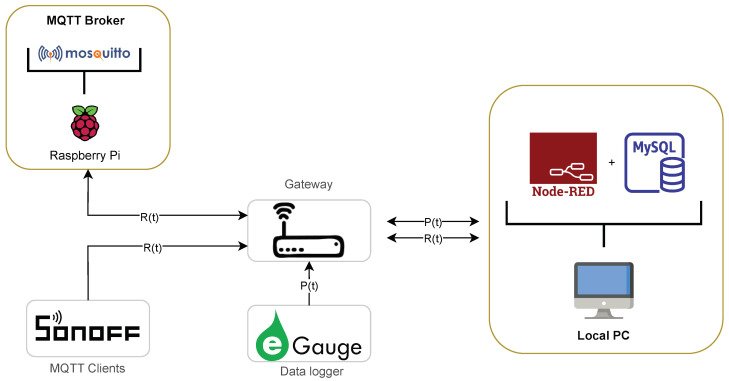
Schematic diagram of the proposed system. Data analytics (DAN) layer components are highlighted in yellow frames.

**Figure 7:**
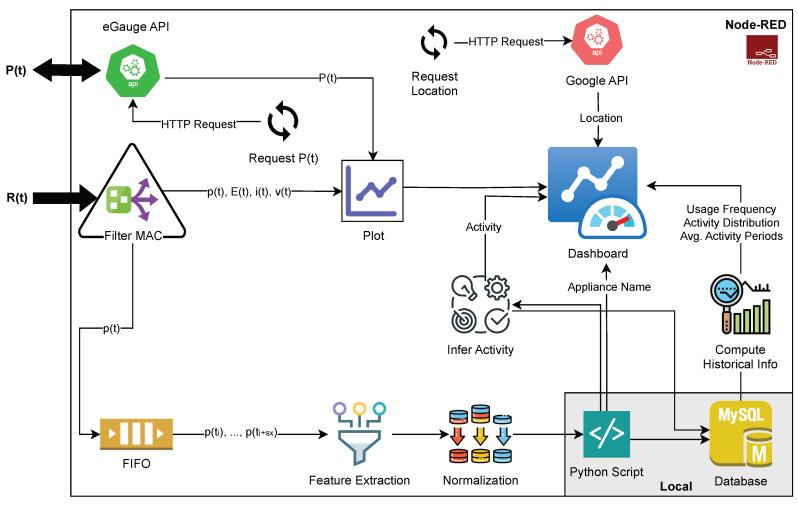
Architecture of the proposed dashboard. The Python script and database elements are highlighted in a different color since they are hosted locally. API: application programming interface; HTTP: hypertext transfer protocol.

In this work, we implemented an edge-based middleware using a Raspberry Pi Model B acting as the MQTT broker, along with a local computer (PC) for subsequent data processing. This architecture is shown in Figure 6, where DAN-layer components are highlighted in yellow frames. Due to smart plug availability in Chile and in order to improve system reliability and effectively monitor low-consumption appliances like the TV and the fridge, we favored intrusive techniques over NILM. The Raspberry Pi is equipped with Eclipse Mosquitto (see https://mosquitto.org/ (accessed on 5 December 2022)) for facilitating communication between the smart plugs and the local PC via the MQTT protocol. On the PC, we developed a dashboard using Node-RED, which provides near real-time information on the electrical consumption of appliances being used and activities performed. Furthermore, a MySQL local database was employed to store historical behavior data, including activity distribution, appliance usage frequency, and average periods of inactivity. These parameters are available per hour, day, or week. The architecture of the dashboard implemented is detailed in Figure 7.

Sonoff devices send messages every 6 s to the broker. The information contained in these messages is represented in Equation (3):

(3) $$\begin{array}{c} r(t)=\{MAC,IP,p(t),E(t),S(t),Q(t),PF(t),i(t),v(t)\};\forall r\in R, \end{array}$$

in which a reading *r* from the set of measurements *R* at time instant *t* contains, in addition to the media access control (MAC) and internet protocol (IP) addresses, the active power p(t), measured in watts (W); the energy consumed E(t), expressed in kilowatt hours (kWh); the apparent power S(t), given in volt-amperes (VA); the reactive power Q(t), measured in volt-ampere reactive (VAR); the power factor PF(t), which is dimensionless and ranges from 0 to 1; the current i(t), given in milliamperes (mA); and the voltage v(t) of the plug, measured in volts (V).

Once a message is received, the system applies filters based on the MAC address to extract specific readings: p(t), E(t), i(t), and v(t). These readings are plotted for each Sonoff device individually. Additionally, the active power readings are accumulated in a first-in first-out (FIFO) queue with a size of ten samples. When the queue is full, an array $\left\{p\left(t_{i}\right),p\left(t_{i+x}\right),p\left(t_{i+2x}\right),\ldots ,p\left(t_{i+sx}\right)\right\}$ of power measurements is created, where *x* represents the sampling frequency (6 s) and *s* corresponds to the window size (10 samples). This array is sent to the feature extractor. The obtained feature vector is normalized and used as input for a ML classifier. The classifier model is stored on the PC and instantiated through a Python script. This approach allows the system to handle multiple queues for different Sonoff devices and enables the parallel instantiation of the classifier model, thereby facilitating the identification of appliances operating simultaneously. Then, each label assigned, which corresponds to the appliance being used, is stored in the MySQL database along with the timestamp of the detection. This allows for the recording and organization of appliance usage information in the database for further analysis and tracking purposes.

In addition, when an appliance is identified, an associated activity is inferred from its usage. Possible activities include thermal comfort (if the heater is turned on), fridge cooling (during the cooling cycles of the minibar), body care (if the hair dryer is in use), water boiling (in case the kettle is switched on), and relaxing (when the TV is detected). Each activity is also stored in the MySQL database, along with its corresponding timestamp. By capturing and organizing this activity information, it becomes possible to analyze the distribution of activities and calculate average inactivity periods. Such analysis can contribute to characterizing a person’s behavior and detecting any unusual deviations from their regular routine. These historical data are obtained as follows:

- Activity distribution: determined by calculating the percentage of time each activity is performed during different time intervals, such as the last hour, last 24 h, and last week.
- Average inactivity periods: calculated by averaging the duration of the inactivity periods during different time intervals, such as the last hour, last 24 h and last week.
- Appliance usage frequency: computed by counting the number of times each appliance is detected during different time intervals, such as the last hour, last 24 h, and last week.

The system also provides additional information such as the total power consumption P(t) and the location. Total power consumption, as shown in Figure 7, is obtained through the eGauge device using an application programming interface (API) provided by the vendor. The system sends requests to retrieve the active power values every second. Similarly, the location information is obtained by making a request to a Google API.

As a result, a user profile is constructed referring to house occupants and based on current total and individual appliance power consumption, as well as the historical values of appliance usage frequency, activity distribution, and average inactivity periods.

### 4.4. Security

Security concerns are conceived as a transversal layer in the proposed architecture. This means that at every layer, we took security measures to ensure the protection of sensitive data, user privacy, and the integrity of the network. As the system is implemented locally, with no internet access, possible attacks include:

- Physical attacks: hardware devices can become damaged or intentionally removed from the plug, thereby hindering the system’s functionality.
- Insecure device configuration: vulnerabilities in device settings can be exploited to gain unauthorized access or disrupt network operations [57].
- Device-to-device interception: even without internet access, an attacker could position between two devices within the LAN and intercept the traffic exchanged between them. This could involve capturing unencrypted communication or attempting to decrypt encrypted traffic if the encryption keys are compromised [57,58].
- Data manipulation: an attacker positioned between two devices can modify the data being exchanged between them. While the modification might not have the same impact as altering internet traffic, it could still lead to unintended consequences within the local network [57].
- Credential harvesting: An attacker might trick users within the local network into revealing sensitive information, such as login credentials, through techniques like phishing or social engineering [57].
- Address resolution protocol (ARP) poisoning: ARP spoofing can still occur within a local network. Attackers can associate their own MAC addresses with IP addresses of legitimate devices, potentially leading to communication redirection or unauthorized access [57].
- Rogue devices: an attacker could set up a rogue device within the network, masquerading as a legitimate device to intercept or manipulate traffic [57].
- Session hijacking: if a device within the network uses sessions for communication, an attacker could attempt to hijack an active session to gain unauthorized access to a device or system [57,58].
- Malware infection: if an infected device is connected directly to the middleware, malware can spread to other devices without internet access [57].

To avoid the aforementioned attacks and ensure integrity of the LAN, especially in the context of smart plugs, MQTT, and Node-RED, we considered the following measures:

- Physical security: ensure physical security on gateways to prevent unauthorized access.
- Strong encryption: use WPA3 encryption for the Wi-Fi network. This provides strong encryption protocols to the data transmitted [59].
- Secure password: set a strong and unique password on all devices and the Wi-Fi network.
- Service set identifier (SSID) hiding: disable broadcasting the network name so that it is not visible to devices scanning for Wi-Fi networks. This adds an extra layer of security by making it less obvious that the network exists [59].
- MAC address filtering: enable MAC address filtering on the gateway to allow only specific devices with approved MAC addresses to connect to the network [59].
- Gateway firmware updates: regularly update the router’s firmware to address security vulnerabilities and ensure the latest security features are in place [59].
- Remote management: disable remote management of the gateway’s settings. This prevents attackers from trying to access its configuration remotely.
- Two-factor authentication (2FA): enable two-factor authentication for accessing the gateway’s settings to add an extra layer of security [59].
- Network segmentation: separate the network into separate virtual area networks (VLANs) for different device types [59].
- Disable unused services: turn off any unnecessary services on the gateway, such as universal plug and play (UPnP) or Wi-Fi protected setup (WPS), as this can introduce potential vulnerabilities.
- TSL/SSL: use encryption (TSL/SSL) for MQTT communication to ensure data confidentiality [59].
- Updates: regularly update and patch software on all components of the middleware to address known vulnerabilities [59].
- Authentication: implement strong authentication and access controls for MQTT [59].
- Educate users: educate users about security best practices and potential threats to prevent social engineering attacks. In this case, it was explained to every staff member in our laboratory.

## 5. Feature Extraction and Classification for Appliance Recognition

  In order to identify the appliances being used by occupants following ILM strategies, in addition to distributed sensing, it is necessary to further process the data received. This processing is known as feature extraction and classification [6,11]. The proposed system acquires the appliance data through the Sonoff Pow R2 devices and forwards this information through Wi-Fi to the middleware, in which a local computer hosts a web application.

We developed a feature extractor which receives an array of power samples and returns a vector of statistical features, as in Equation (4):

(4) $$\begin{array}{c} \mathrm{vector}=[{}^{\prime }\min^{\prime },{}^{\prime }\max^{\prime },{}^{\prime }{\mathrm{mean}}^{\prime },{}^{\prime }{\mathrm{std}}^{\prime },{}^{\prime }{\mathrm{skew}}^{\prime },{}^{\prime }{\mathrm{kur}}^{\prime },{}^{\prime }{\mathrm{var}}^{\prime },{}^{\prime }{\mathrm{mad}}^{\prime },{}^{\prime }\mathrm{above}\_{\mathrm{mean}}^{\prime },{}^{\prime }{\mathrm{zeros}}^{\prime }], \end{array}$$

in which each element in the array corresponds to a specific featured extracted from the data, including minimum (‘min’), maximum (‘max’), mean (‘mean’), standard deviation (‘std’), skewness (‘skew’), kurtosis (‘kur’), variance (‘var’), mean absolute deviation (‘mad’), count above the mean (‘count_mean’), and count of zero values (‘zeros’). The function to extract and calculate the features is detailed in Algorithm 1. The proposed function extracts statistical features using a sliding window approach, storing elements in a FIFO queue. It iterates over the data and calculates features given in Equation (4) within each window. The vector obtained is stored in an array. This function handles different window size options and considers padding if necessary. However, we used a window size of 10 and padding as recommended in [11]. The proposed feature extractor efficiently processes the data and provides a comprehensive set of features for classification.

To properly handle highly varying magnitudes negatively impacting on classification, we performed feature scaling following the Scikit-Learn’s MinMaxScaler function, shown in Equation (5):

(5) $$\begin{array}{c} \mathrm{Scaler}\left(x\right)=\frac{x-\min(x)}{\max(x)-\min(x)} \end{array}$$

where max(x) and min(X) are the maximum and the minimum values of the feature, respectively. If feature scaling is not performed, then the ML model tends to give more weight to larger values, and to consider smaller values as the lower values, regardless of their units.

**Figure 8:**
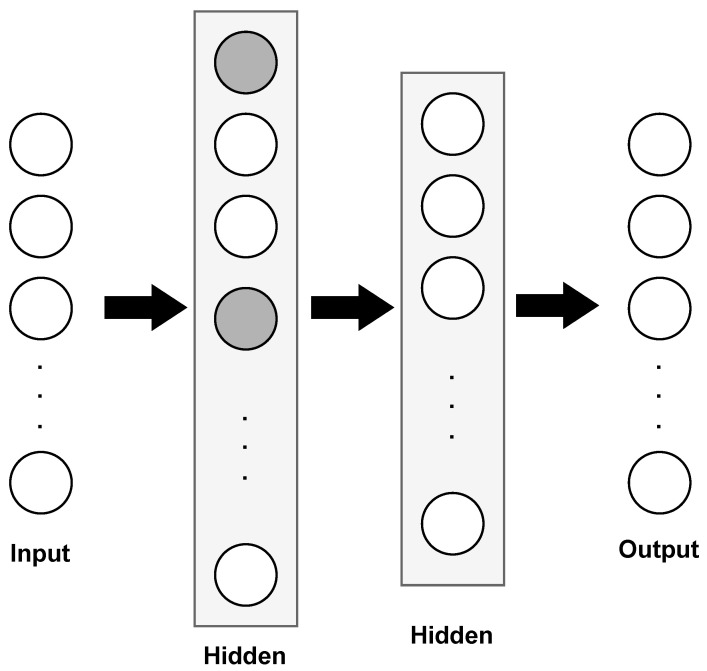
Structure of the proposed machine learning classifier. The model has two hidden layers of 500 and 100 units, respectively. Dropout is represented by turning off units (gray color) in the first hidden layer. Neurons not affected by dropout are represented in white color.

The proposed ML classifier is shown in Figure 8. It follows a feed-forward neural network architecture (FFNN) built in the Keras framework with a Tensorflow backend. We created the model as sequential, meaning that layers were added sequentially. The first layer is a fully connected layer (dense) with 500 unit/neurons. We added a dropout layer after the dense layer to help prevent overfitting by randomly setting a fraction of units to 0 during training. The second layer is another fully connected layer of 100 units, and the final is a fully connected layer with the number of units corresponding to the number of classes in the target variable. As our testbed includes five appliances (five classes), then the final layer has five units, this being the output of the model. In operation, each neuron of the proposed model computes a weighted sum of its inputs, adds a bias term, applies an activation function, in this case ReLU (Equation (6)), to introduce non-linearity, and passes the result to the next layer. The weights and biases are adjusted during training to capture complex relationships between the input data and the desired output. Dense layers enable neural networks to learn and model intricate patterns, making them powerful tools for solving a wide range of tasks, such as appliance recognition.


**Algorithm 1:** Function to view features in a window.
1:create a FIFO queue with a maximum size of 10 if it does not exist.2:push new data to the FIFO queue.3:**if** (the queue is full) **then**4:    stride = 105:    window_size = 106:    activation_threshold = 37:    mode = ‘padding’8:    zeros = 09:    above_mean = 010:    **for** each element in FIFO **do**11:        **if** value ≥ activation_threshold **then**12:             Store activation values in a list.13:        **else**14:             zeros += 1 {Count of zero values inside the window.}15:        **end if**16:    **end for**17:    min = min(activations) {Minimum activation value.}18:    max = max(activations) {Maximum activation value.}19:    mean = sum(activations)/ count(activations) {Mean activation value.}20:    sum_of_squared_deviations = reduce(map(activations, x ⇒ (x − mean)${}^{2}$), 0, (sum, deviation) ⇒ sum +    deviation)21:    mean_squared_deviation = sum_of_squared_deviations/count(activations)22:    std = square_root(mean_squared_deviation) {Standard deviation of activation values.}23:    **if** (activations has at least 3 values) **then**24:         sum_of_cubed_deviations = reduce(map(activations, x ⇒ (x − mean)${}^{3}$), 0, (sum, deviation) ⇒ sum +         deviation)25:         skew = sum_of_cubed_deviations/(activations.length ∗ std${}^{3}$) {Skewness of activation values.}26:    **else**27:         skew = 028:    **end if**29:    **if** (if activations has at least 4 values) **then**30:         sum_of_fourth_power_deviations = reduce(map(activations, x ⇒ (x − mean)${}^{4}$), 0, (sum, deviation) ⇒         sum + deviation)31:         kur = sum_of_fourth_power_deviations/(activations.length ∗ std${}^{4}$) {Kurtosis of activation values.}32:    **else**33:         kur = 034:    **end if**35:    var = std/mean {Variance of the activation values.}36:    absolute_deviations = map(activations, x ⇒ abs(x − mean))37:    mad_sum = reduce(absolute_deviations, 0, (sum, deviation) ⇒ sum + deviation)38:    mad = mad_sum/count(activations) {Mean absolute deviation of the activation values.}39:    **for** each value x in activations **do**40:         **if** x > mean **then**41:              above_mean += 1 {Values above the mean activation.}42:         **end if**43:    **end for**44:    vector = [min, max, mean, std, skew, kur, var, mad, above_mean, zeros]45:    empty activation list.46:
**end if**
47:shift the queue.48:**return** vector



The ReLU activation function is given by Equation (6):

(6) $$\begin{array}{c} f\left(x\right)=\left\{\begin{array}{cc} x, & \mathrm{if}x\geq 0\\ 0, & \mathrm{otherwise} \end{array}\right. \end{array}$$

To compile the model, we used the categorical cross-entropy loss function, which is commonly used for multiclass classification tasks. This function is defined as in Equation (7):

(7) $$\begin{array}{c} \mathrm{Categorical}\mathrm{Cross}-\mathrm{Entropy}\mathrm{Loss}=-\sum_{i=1}^{C}y_{i}\log\left(p_{i}\right), \end{array}$$

where *C* is the number of classes, $y_{i}$ represents the true label (ground truth) for the i-th class and $p_{i}$ the predicted probability for the i-th class outputted by the model. The loss function calculates the logarithms of the predicted probabilities and multiplies them with the true labels. By summing these values over all classes and taking the negative, the loss penalizes larger discrepancies between the predicted and true probabilities.

It is important to remark that we selected the values of window size, number of layers, and neurons, along with the type of model, based on the best results obtained in [10,11].

## 6. Results

In order to prove the reliability of the proposed system, we performed a series of experiments. The first step was to train the ML classifier. In this regard, we collected two weeks of data from the five appliances and stored them in comma-separated value (CSV) files. A summary of the collected data is shown in Table 3. It highlights the number of instances collected (100,582), the format of the data (a JSON including the timestamp and the values of r(t) defined in Equation (3)), and the number of missing values (two). From the JSONs we extracted the timestamp and r(t) and stored them in the CSV file for further use.

We applied the sliding window approach described in Algorithm 1 for the active power samples and formed a statistical features dataframe to input to the model. Considering that most smart plug devices available on the market measure active power, we specifically focused on this parameter to build a standardized system that can be applied to various smart plug brands. The advantage of our approach is that the model relies on statistical features derived from the active power readings, rather than being dependent on specific device characteristics or proprietary features. Therefore, if plugs different from Sonoff Pow R2 devices are used, the model can still perform classification accurately, ensuring its applicability and usefulness in various settings and scenarios.

### 6.1. Training Results

**Figure 9:**
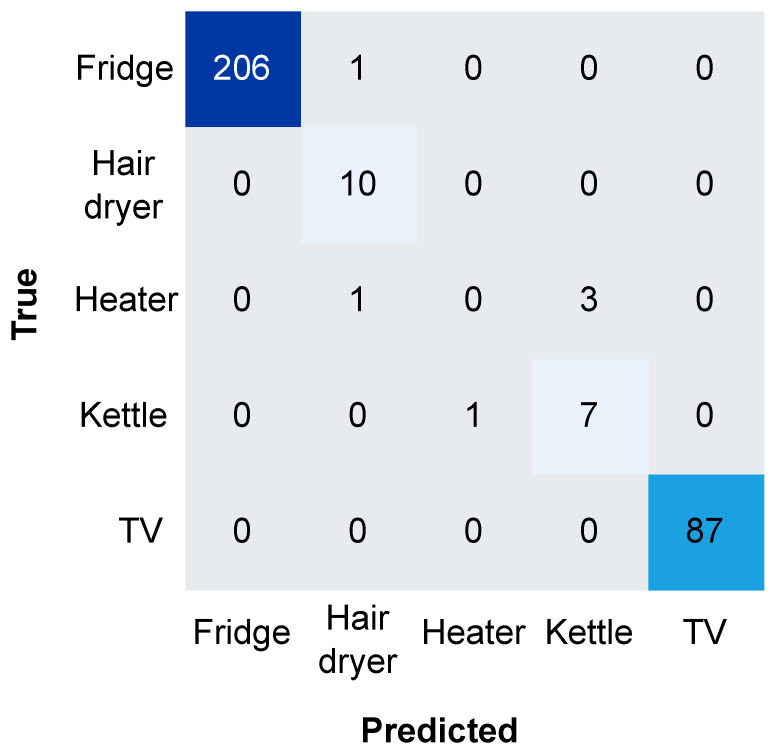
Confusion matrix obtained with the test set for the proposed classifier. Colors represent the total of correctly classified samples for each class, from gray (none or few samples) to dark blue (more than 100 samples).

From the two-week dataset, we used 80% for training, 10% for validation, and the remaining 10% for testing. Figure 9 shows the confusion matrix obtained with the test set. As can be seen, significant class imbalance negatively impacts the model’s performance. There is a considerable difference in the number of samples of the fridge and TV compared with the rest of the appliances. This happens due to the variations in the frequency in appliance usage throughout the day. For example, certain appliances like the TV may be used more frequently than others such as the hair dryer, depending on the occupants’ habits and routines. A special case is the fridge, since it is continuously connected and automatically goes through cooling cycles, not reflecting user behavior. However, monitoring permanent loads such as fridges may be useful for other purposes, such as detecting malfunctions or failures in the appliance. For this reason we considered the fridge for classification but excluded it from activity analysis concerns.

The model accurately classifies the majority of classes, fridge and TV, having a single misclassification between the two. For minority classes such as the heater, the situation is the opposite, no samples are correctly classified in this case.

Table 4 shows the classification metrics for the five appliances using the proposed model. The precision metric measures the correctly classified proportion for each appliance. The recall metric measures the proportion of correctly identified instances out of the total instances for each appliance. The F1 score combines precision and recall, providing a balance measure of the model’s performance for each appliance. The results show the negative impact of the imbalanced dataset on the model, with a significant difference in performance between the majority and minority classes. For example, the fridge obtains a 100% precision while the heater obtains 0%. In the case of the kettle, 70% of the positive predictions are correctly classified. Although the heater is a multi-state appliance, most of the time its operation switches between only two levels of power consumption, causing the ML model to misclassify it on three occasions as a kettle, the operation of which transitions between on and off, thus, decreasing its classification precision. However, the model demonstrates an accuracy of 98%, reflecting the correctly classified instances across all appliances but not fully accounting for the class imbalance in the data. The addition of the Cohen’s kappa coefficient provides a more comprehensive understanding of the model’s behavior by normalizing the classification accuracy based on the class distribution. This measure was calculated as 96%, indicating a strong level of agreement between the predicted and actual classifications.

Once the model was trained, it was stored and instanced in a Python script for further integration with the Node-RED environment.

### 6.2. Real-Time Operation

**Figure 10:**
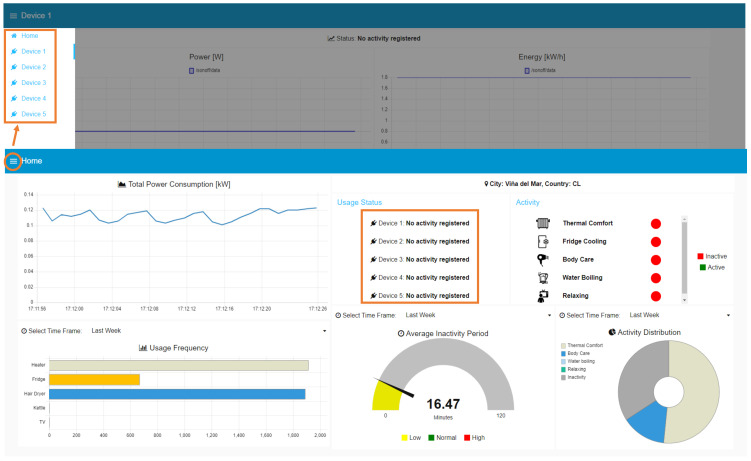
Web interface of the proposed SEDAR. Main dashboard side menu and non-activity status are highlighted in orange frames.

The web interface comprises six views, one for each individual Sonoff device and the main dashboard. Each device view displays the status, corresponding to the name of the appliance in use, along with the p(t), E(t), i(t), and v(t) readings. If no appliance is detected in a given plug, `’no activity registered” will be displayed instead. Examples of these situations are shown in Figure 10. The user can switch between the statuses of the five devices, to receive a more complete description of the appliance’s consumption or obtain a summarized version along with detailed activity information in the main dashboard.

In the case of the main dashboard, shown in Figure 10, it provides an overview of the occupant’s profile, including the location, total power consumption, a summary of the status of each Sonoff device, the ongoing activities, the appliance usage frequency, the average inactivity periods, and the activity distribution. This comprehensive display allows users to easily access and analyze the collected data and gain insights into the occupant’s energy consumption patterns and daily activities. The appliance usage frequency, average inactivity periods, and activity distribution complement the occupant’s profile. In Figure 10, these parameters are shown for the last 24 h, however, the system allows users to obtain historical parameters for the last hour and the last week as well.

**Figure 11:**
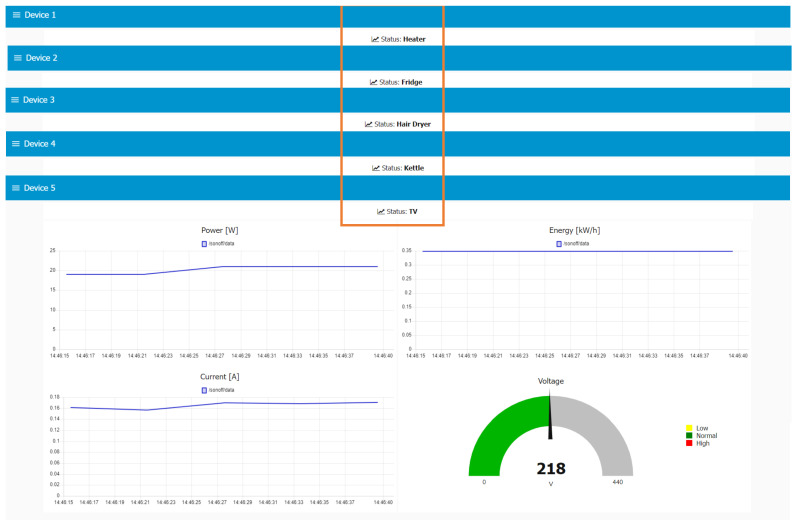
Devices view, showing all appliances correctly classified. Statuses are highlighted in orange frames.

All appliances are successfully identified by the system, including those representing the minority classes, such as the heater, with no correctly classified samples in the test set. Figure 11 shows screenshots of the Sonoff devices view recognizing each of the appliances. This proves the relative reliability of metrics such as precision and recall, commonly used in classification problems, which may be affected by class imbalance.

**Figure 12:**
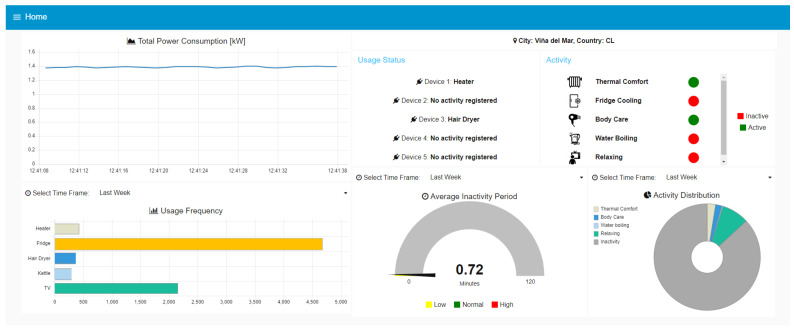
Main dashboard screenshot during simultaneous activity detection.

Figure 12 shows the simultaneous detection capability of the system, highlighting one of its key advantages. In this particular scenario, both the heater and the hair dryer are active, indicated by Sonoff devices 1 and 3, respectively. This simultaneous detection allows us to infer activities related to body care, such as hair drying, while also ensuring thermal comfort by using the heater.

The ability to detect and identify multiple appliances at the same time provides valuable insights into the occupant’s behavior. Capturing and analyzing such information enhances the system’s usefulness in various contexts, such as energy management, behavior monitoring, and anomaly detection.

**Figure 13:**
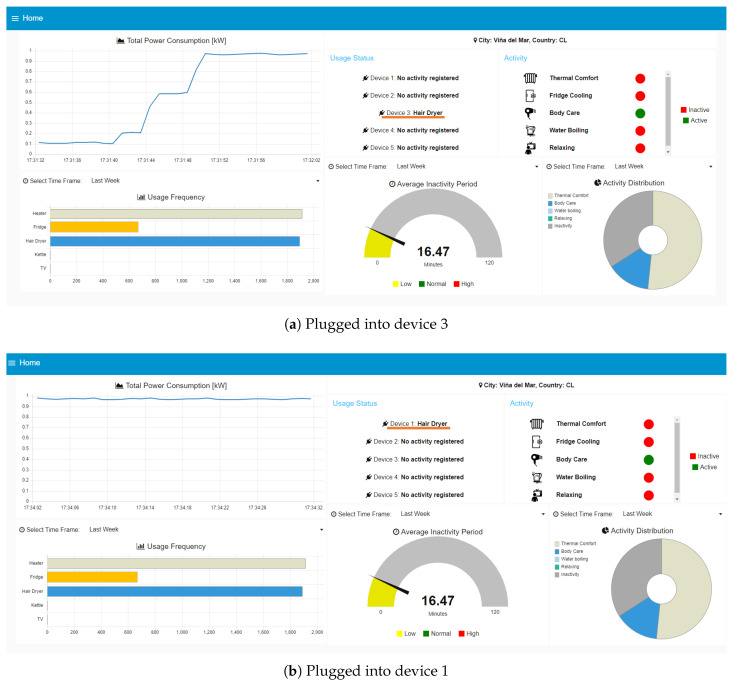
Screenshots of the main dashboard showing the hair dryer being moved from device 3 to device 1. Appliance name is underlined in orange.

Another important capability of the system is its flexibility, allowing any appliance to be connected independently of the plug and still being able to recognize it. In Figure 13, the hair dryer was moved from Sonoff 3 to 1. Despite this change, the system successfully identified the hair dryer in both cases. This flexibility is achieved through the utilization of active power statistical features rather than relying on specific plug or device characteristics.

## 7. Discussion and Limitations

Compared to prior state-of-the-art family-oriented approaches, such as [8,25], the proposed SEDAR had an improved accuracy, achieving a 96% Cohen’s kappa coefficient regardless of the significant class imbalance in the dataset. Furthermore, our system is capable of detecting and identifying multiple appliances at the same time, providing valuable insights into the occupant’s behavior. Other solutions, such as [26,27,28,29], have either limited explainability or lack of a practical implementation of their system. The proposed SEDAR, on the other hand, provides reliable insights and represents an innovative approach to appliance and activity recognition, which successfully identifies the appliances in use and accumulates historical activity data for further processing. Using advanced techniques in ML and data processing, our system offers a robust and reliable solution to understand and monitor appliance usage in a home setting. Other approaches, such as [1,22,23,24], focused on medical-oriented services, mainly following NILM techniques, which can lead to unreliability in the results obtained since this method is based on the smart meter signal. The aggregated power consumption signal can be very noisy, only allowing major appliances to be detected, i.e., those with higher electrical consumption. In addition, access to smart meters is limited in many countries, including Chile, due to regulation issues. Therefore, the proposed system can impact significantly in a future massive deployment, increasing acceptability, since smart plugs can be installed throughout the house and appliances are not required to be attached to a specific plug, adding flexibility to the proposed system and making the house smart.

One of the key strengths of the proposed system lies in its ability to accurately identify specific appliances in near real time. However, some limitations exist when working at a lower resolution. For example, varying the stride and queue size can decrease the recognition time, since with the current configuration ten samples need to be collected before extracting features and classifying. As the sampling frequency is 6 s, the appliance will be detected 1 min after it has been turned on. Similar happens when the appliance is turned off, there will be a 1 min delay before the system returns to “no activity registered” status. This delay can be handled by decreasing the window size [11]. In addition, we have added a second activation threshold for activity inference. This way, a double condition needs to be accomplished in addition to detecting a given appliance: the active power has to be above a certain level. This increases the recognition time for ’off’ states.

Through the utilization of sensor data, we can distinguish between different appliances and capture their usage patterns with high precision. This capability opens up a range of possibilities for energy management, load balancing, and appliance-specific optimization strategies. However, the performance encountered some limitations. For multi-state appliances, such as the hair dryer and the electric heater, transitions were always misclassified. In certain states, these appliances have similar power consumption, therefore, the model will wrongly assign the label. To solve this issue, various modifications can be performed. A more balanced dataset is necessary, which captures the variations in multi-state appliances. Other features need to be explored that help the system discriminate when the active power is the same for different multi-state devices. In addition, by making the system remember past states, the transitions issue may also be solved.

The proposed system is oriented to elderly people living alone. It remains a challenge for a multi-user-oriented solution. However, the system goes beyond immediate recognition by accumulating historical data. By continuously capturing and analyzing activity patterns over time, we enable deeper insights into household dynamics, energy consumption patterns, and occupant behaviors. These accumulated data serve as a valuable resource for energy auditing, behavioral analysis, and the development of personalized energy-saving recommendations. More complex scenarios need to be explored, including forecasting capabilities for reliable anomaly detection.

Overall, the system remains non-obtrusive to occupants. It seamlessly integrates into their daily routine without requiring any additional effort or modification. The system operates transparently in the background, continuously monitoring and identifying appliances without interfering with their normal usage.

## 8. Conclusions

In this work, a smart energy data with activity recognition system is designed and implemented towards enabling remote elderly care. The system has a three-layer architecture, namely, data acquisition, communication network, and data analytics. Sonoff Pow R2 devices were used to send a message every 6 s to a Raspberry Pi acting as an MQTT broker. In a Node-RED environment, these messages are processed allowing appliances in use to be identified and inferring an activity from them. An ML classifier receives a vector of active power features and returns a label corresponding to the appliance name. Historical data are available through communication with a MySQL database. The occupant’s profile, along with near real-time data, is accessible to users such as caregivers and people monitoring the occupants through a user-friendly web interface. This interface provides valuable information about appliance usage, activity patterns, and occupant behavior.

One of the main advantages of the system is its non-obtrusiveness. It seamlessly integrates into the living environment without imposing any significant changes in occupant’s daily routines. Additionally, flexibility and versatility, as it can adapt to different appliance types and is compatible with existing infrastructure. The classifier model achieves a 96% Cohen´s kappa coefficient, demonstrating strong accuracy, even in scenarios involving simultaneous operations. Nonetheless, there are still limitations that need to be addressed. Due to the sampling frequency of 6 s, the system experiences a delay of approximately 1 min in recognizing whether an appliance has been turned on or off. Moreover, when it comes to multi-state appliances, the system consistently misclassifies transitions.

The proposed system can positively impact ambient assisted living and energy efficiency, being a complementary technology to remotely monitor the well-being of seniors living alone, and accounting for the energy consumption of the household, which allows its future integration with an HEMS. Future work will be oriented to refine and expand the capabilities of the proposed system by exploring other features and integrating forecasting. In addition, special algorithms and more complex strategies to deal with class imbalance will be developed. This represents a step further in developing sustainable and intelligent homes. 

## Figures and Tables

**Table 1:** Summary of previous research work.

Reference	Type	Wearable Sensors	Camera	ILM	NILM	Target Application
[1]	Technical	X	X	X	✓	Dementia
[25]	Technical	X	X	X	✓	Anomalous behavior
[31]	Survey	✓	X	X	X	Vital signs monitoring
[23]	Technical	X	X	✓	X	Accident prevention, fall detection
[20]	Technical	✓	X	X	X	Rehabilitation
[19]	Survey	✓	X	X	X	Behavioral patterns
[32]	Survey	✓	✓	✓	X	Anomalous behavior
[22]	Technical	X	X	X	✓	Parkinson’s Disease
[33]	Survey	X	X	✓	X	Elderly care
[18]	Survey	✓	X	X	X	Rehabilitation
[34]	Survey	✓	X	✓	X	In-home monitoring
[26]	Technical	X	X	✓	X	Behavioral patterns, comfort
[35]	Technical	X	X	✓	X	Elderly care
[27]	Technical	X	X	✓	X	Anomalous behavior
[36]	Technical	✓	X	X	X	Elderly care
[37]	Technical	✓	X	✓	X	Remote monitoring for people in rural areas
[24]	Technical	X	X	✓	X	Elderly care
[28]	Technical	X	X	✓	X	Surveillance, in-home monitoring
[38]	Technical	✓	X	X	X	Exertion recognition, asthenia
[39]	Technical	X	X	✓	✓	Not specified
[21]	Technical	✓	✓	X	X	In-home monitoring
[40]	Technical	✓	X	X	X	In-home monitoring
[41]	Survey	X	X	X	✓	AAL
[42]	Survey	✓	X	X	✓	In-home monitoring
[43]	Survey	✓	✓	X	X	In-home monitoring
[44]	Survey	X	X	✓	X	In-home monitoring
[3]	Survey	X	X	✓	X	In-home monitoring
[29]	Technical	X	X	✓	X	In-home monitoring
[45]	Technical	X	X	✓	X	Recommendations
[46]	Technical	X	✓	X	X	Activity Recognition
[47]	Technical	X	X	✓	X	Activity Recognition
This work	Technical	X	X	✓	X	In-home monitoring, elderly care, HEMS integration

X: Not considered. ✓: Considered.

**Table 2:** List of appliances considered.

Appliance	Brand	Model	City	Country
Kettle	Hamilton Beach	40987-CL	Valparaiso	Chile
TV	LG	24TL520S-PS	Valparaiso	Chile
Hair dryer	Siegen	SG-3049	Valparaiso	Chile
Minibar	Nex	CR-52	Santiago	Chile
Electric heater	Ufesa	RD-1500D	Santiago	Chile

**Table 3:** Summary of the collected data.

Instances	Format	Missing Values
100,582	JSON(timestamp, r(t) (Eq.)) [3]	2

**Table 4:** Classification metrics.

Appliance	Precision	Recall	F1 Score
Fridge	1.00000	0.99517	0.99758
Hair dryer	0.83333	1.00000	0.90909
Heater	0.00000	0.00000	0.00000
Kettle	0.70000	0.87500	0.77778
TV	1.00000	1.00000	1.00000
Accuracy	0.98101		
Cohen’s kappa	0.961651		

## Data Availability

Not applicable.

## Abbreviations

The following abbreviations are used in this manuscript:

2FA	Two-factor authentication
AAL	Ambient assisted living
ADLs	Activities of daily living
AI	Artificial intelligence
API	Application programming interface
ARP	Address resolution protocol
CSV	Comma-separated value
DAQ	Data acquisition
DAN	Data analytics layer
EVs	Electric vehicles
FIFO	First-in first-out queue
GES	Explicit health guarantee
HEMS	Home energy management system
HVAC	Heating, ventilation, and air conditioning
HTTP	Hypertext transfer protocol
IDS	Intrusion detection system
ILM	Intrusive load monitoring
IoT	Internet of things
IR	Infrared
LAN	Local area network
ML	Machine learning
MQTT	Message queue telemetry transport protocol
NCTS	Non-contact triboelectric sensors
NILM	Non-intrusive load monitoring
PD	Parkinson’s disease
PLCs	Power line communications
RFID	Radio-frequency identification
SEDAR	Smart energy data with activity recognition
SSL	Secure socket layer
TLS	Transport layer security
TV	Television set
UTFSM	Universidad Técnica Federiso Santa María
VLAN	Virtual local area network
WAN	Wide area network
WPA3	Wi-Fi Protected Access 3
